# What are the limits to feed intake of broilers on bulky feeds?

**DOI:** 10.1016/j.psj.2020.11.008

**Published:** 2020-11-19

**Authors:** James Taylor, Panagiotis Sakkas, Ilias Kyriazakis

**Affiliations:** ∗ Agriculture, School of Natural and Environmental Sciences, Newcastle University, Newcastle on Tyne NE1 7RU, UK; † CCPA Group, Z.A. du Bois de Teillay, Janzé 35150, France; ‡ Institute for Global Food Security, Queen's University, Belfast BT7 1NN, United Kingdom

**Keywords:** bulk, broiler, feed intake, gastrointestinal tract, sugar beet pulp

## Abstract

The view that genetic selection for carcass yield has limited the size of the gastrointestinal tract (GIT) of modern broilers has sparked concerns that their capacity to cope with energy dilution or bulk is also limited. We investigated the capacity of male Ross 308 broilers to deal with increasing levels of bulk and aimed to identify a feed bulk dimension responsible for limiting feed intake (FI). About 528 day-old broilers were allocated to 48 pens and offered a common starter feed until day 8, and 1 of 7 feeds from day 8 to 36 of age: a basal control (B), which was diluted to 3 levels (15, 30, or 45%) with either oat hulls (OH) or sugar beet pulp (SBP). Feed intake was measured daily and birds were dissected for GIT measurements at day 15, 22, and 36. Feed intake increased in birds offered OH15 (135 g/d), OH30 (140 g/d), and SBP15 (138 g/d) compared with birds offered the B feed (106 g/d; SEM 2.4). By increasing FI, birds were able to compensate for the lower energy content of their feeds. The greatest increase in FI was seen on OH30: its energy content (2,273 kcal/kg) was 26% lower than the B feed (3,081 kcal/kg). There was evidence of adaptation on the bulky feeds, as during the last week only birds on SBP45 were limited in FI and performance. The relative weights of the GIT were greater in the SBP than OH series, suggesting that the former needed to accommodate a higher bulk intake. For the OH series the increase in the relative GIT weights was confined to the gizzard and small intestine; whereas for the SBP series, the increase was extended to proventriculus and large intestine. Because only SBP45 was limiting FI, we were unable to identify a bulk dimension to be used to predict FI. Our data reject the suggestion that modern broilers have a reduced ability to cope with reductions in feed energy content.

## Introduction

There is growing interest in using alternative feed ingredients in livestock production due to the competition between food and feed, the need to reduce the environmental impact of livestock production and reduce the costs associated with importing non-native ingredients (Madeira et al., 2017; Tallentire et al., 2018b). As far as poultry are concerned, such alternative ingredients include co- and by-products of food production which are not desirable to humans, such as wheat middlings, rice bran, palm kernel meal (Ravindran, 2013), and oat hulls (OH) (Scholey et al., 2020). Such ingredients are lower in energy (Robert et al., 1997; Sundu et al., 2006) and possess a high bulk content than traditional ingredients due to their physicochemical properties, such as water-holding capacity (WHC) (Jiménez-Moreno et al., 2009). Studies on pigs have shown that properties of bulk, such as “fibre” content and WHC can accurately predict feed intake capacity on bulky feeds (Tsaras et al., 1998; Ndou et al., 2013). Such bulk characteristics may have the potential to predict the feed intake of poultry (Kyriazakis et al., 1994). However, there is a dearth of information as to whether this is indeed the case.

At the same time, there have been increased concerns about the ability of modern poultry, especially broiler genotypes to cope with bulk intake and a reduction in feed energy content (Mbajiorgu et al., 2011; Gous, 2013; Classen, 2017; Scholey et al., 2020). Historically, there has been an abundance of evidence showing that the GIT of broilers can adapt and increase in size relative to BW when the birds are offered feeds with bulky ingredients (Griffiths et al., 1977; Deaton and Lott, 1985; Leeson et al., 1996a). More recently, however, Linares and Huang (2010) observed a compensatory increase in feed intake when dietary energy was reduced by 5%; however, when energy was reduced by 10%, there was no further increase in feed intake and the performance of the birds was penalized. Classen (2017) has suggested that intensive artificial selection has caused the modern broiler to lose the ability to adjust feed intake in response to reductions in dietary energy and that care needs to be taken when including alternative ingredients in broiler diets. Several studies showing that broilers are unable to adjust feed intake when offered feeds of different nutrient compositions, support this suggestion (Sahraei and Shariatmadari, 2007; Swennen et al., 2007; Latshaw, 2008; Delezie et al., 2010; Cho, 2011; Krás et al., 2013). Pym (2005) has suggested that broilers which are selected for increased appetite or growth rate alone may be eating to GIT capacity, whereas birds selected for improved nutrient utilization retain the ability to modify feed intake in response to nutrient composition.

The ability for animals to cope with feeds of a high bulk content is constrained by the physical capacity of the GIT (Kyriazakis and Emmans, 1995). Tallentire et al. (2018a) suggested that the capacity of the GIT in broilers is nearing its biological limits and as such, so is the capacity for maximum daily feed intake. An explanation for this has been suggested to be that selection for increased carcass yield has occurred at the expense of the size of the GIT (Mitchell and Smith, 1991; McEntee et al., 2003; Rougière et al., 2009; De Verdal et al., 2010; Lumpkins et al., 2010; De Verdal et al., 2011; Alshamy et al., 2018). The opposite view has also been put forward (Zuidhof et al., 2014), as there is evidence to suggest that the GIT of modern broilers has increased compared with a heritage line when GIT measurements were expressed per unit of BW (Schmidt et al., 2009). As the interest in alternative, bulky ingredients grows, it is important to understand the physical limits of the broiler GIT and their ability to cope with bulky feeds.

The aim of this experiment was 2-fold: 1) to assess the ability of a modern broiler strain to cope with feeds which were progressively diluted with the bulky ingredients OH or sugar beet pulp (SBP). We hypothesized that broilers offered feeds diluted with SBP will be limited in their performance further than those offered feeds diluted with the same amount of OH due to differences in the bulk properties of the 2 ingredients, such as the WHC; 2) to identify a bulk property which might be responsible for limiting feed intake and use it for future predictions of feed intake on bulky feeds.

## Materials and methods

### Feed Formulation

All birds were fed the same conventional starter feed from day 0 until day 8 of age. They were offered the experimental feeds from day 8 to 36 of age (Table 1). Three grower feeds were formulated (Table 1): a basal feed (B), which was subsequently diluted with 45% of either OH or SBP to produce 2 bulky feeds. These diluents were selected because of their stark differences in physicochemical properties, such as the WHC, which is higher in SBP than in OH, whereas the converse is the case for their crude fibre contents (González-Alvarado et al., 2010). Each bulky feed had the same calculated digestible protein-to-AME ratio, and all other nutrient-to-AME ratios were the same as in the basal feed. Nutrient ratios were maintained by increasing or decreasing synthetic amino acids, limestone, and monocalcium phosphate inclusion, where appropriate.

**Table 1:** Ingredient, calculated, and analyzed chemical composition of the grower foods offered from day 8 to day 36 of age to broiler chickens.

Ingredients (%)	B	OH15	OH30	OH45	SBP15	SBP30	SBP45
Ground maize	10.0	8.50	7.00	5.50	8.50	7.00	5.50
Ground wheat	54.4	44.1	33.8	23.6	44.2	34.0	23.8
Soybean meal (48% CP)	22.0	19.0	15.9	12.9	19.0	15.9	12.9
Soybean meal (70% CP)	1.00	1.33	1.67	2.00	1.33	1.67	2.00
Full fat soya	5.00	4.62	4.23	3.85	4.62	4.23	3.85
Soya oil	3.30	3.03	2.77	2.50	3.03	2.77	2.50
Oat hulls (OH)	-	15.0	30.0	45.0	-	-	-
Sugar beet pulp (SBP)	-	-	-	-	15.0	30.0	45.0
Limestone	1.45	1.27	1.10	0.93	0.97	0.48	-
Monocalcium phosphate	1.25	1.12	1.00	0.87	1.35	1.46	1.56
L-Lysine HCL	0.30	0.40	0.51	0.61	0.42	0.54	0.67
DL-Methionine	0.35	0.30	0.26	0.21	0.32	0.28	0.25
L-Tryptophan	-	0.01	0.02	0.03	0.02	0.04	0.06
L-Threonine	0.15	0.20	0.25	0.30	0.21	0.27	0.34
L-Arginine	-	0.12	0.23	0.35	0.13	0.26	0.39
Valine	-	0.09	0.17	0.26	0.08	0.17	0.25
Iso-leucine	-	0.08	0.16	0.24	0.09	0.17	0.26
Salt	0.25	0.22	0.19	0.16	0.22	0.18	0.15
Sodium bicarbonate	0.15	0.14	0.13	0.12	0.13	0.11	0.08
Vitamin and mineral premix	0.40	0.40	0.40	0.40	0.40	0.40	0.40
Titanium dioxide	0.50	0.50	0.50	0.50	0.50	0.50	0.50
Ronozyme^1^	0.02	0.02	0.02	0.02	0.02	0.02	0.02
Total	100	100	100	100	100	100	100
Chemical composition (%)^2^							
Metabolizable energy (kcal kg^−1^) (calculated)	3,009	2,759	2,502	2,244	2,916	2,815	2,713
Crude protein (CP)	25.4	23.3	21.2	19.1	23.6	21.7	19.8
Digestible crude protein (dCP, calculated)	22.0	19.9	17.9	15.9	20.9	19.9	18.9
Lysine (calculated)	2.01	1.84	1.67	1.49	1.94	1.87	1.80
Digestible lysine	1.88	1.74	1.56	1.39	1.82	1.79	1.69
Threonine (calculated)	1.30	1.19	1.07	0.96	1.26	1.21	1.17
Ether extract (oil A)	6.39	6.04	5.16	5.19	5.50	5.35	5.96
Total oil (oil B)	6.94	6.77	5.96	5.66	6.04	6.11	6.70
Ash	6.20	5.40	5.30	5.40	7.90	7.90	9.00
Calcium (calculated)	0.98	0.89	0.81	0.73	0.97	0.97	0.97
Available phosphorus (calculated)	0.41	0.36	0.32	0.27	0.42	0.43	0.44
Crude fibre	2.70	5.90	8.70	10.7	5.80	6.20	6.50
Neutral detergent fibre	9.70	16.2	24.2	28.5	14.7	15.8	15.7
Acid detergent fibre	3.01	7.48	12.2	13.3	7.57	9.96	8.34
Acid detergent lignin	0.73	1.69	2.29	2.49	1.24	1.26	1.27
Insoluble dietary fibre	10.2	18.4	26.0	37.5	19.3	22.4	30.5
Soluble dietary fibre	1.60	2.40	2.00	1.30	4.30	6.20	8.60
Total dietary fibre	11.8	20.8	28.0	38.8	23.6	28.6	39.1
Feed density (g/mL)	1.28	1.21	1.29	1.38	1.39	1.51	1.66
Water-holding capacity (g/g DM)	2.65	2.53	2.74	3.09	3.46	4.73	6.01

The basal (B), high-density food was diluted with 15, 30, or 45% of either oat hulls (OH) or sugar beet pulp (SBP).

1 Blend of amylase and beta-glucanase.

2 Analyzed composition unless otherwise stated.

Mixtures of the basal feed and the 2 bulkiest feeds were created to produce 3 dilutions of bulky feeds for each diluent. The first mixture was 1-part basal and 2-parts bulky feeds to produce a 30% dilution of the basal feed, and the second mixture was 2-parts basal and 1-part bulky feed to produce a 15% dilution, so all resulting feeds had the same nutrient-to-AME ratios. The experimental feeds were offered in pellet form. Each of the bulky feeds and their dilutions were replicated in 7 pens, while there were 6 replicate pens for the basal treatment.

### Experimental Design and Birds

All procedures were conducted under the UK Animals (Scientific Procedures) Act 1986 and were approved by the Animal Welfare and Ethical Review Body of Newcastle University no. 7332/2018. A total of 528 male Ross 308 chicks were obtained at day 0 of age from a commercial hatchery and were housed in a thermostatically controlled building with 48 pens, each with an area of 0.85 m^2^. Pens were equipped with feeders and drinkers, with wood shavings used as litter at a depth of 5 cm. All birds were wing-tagged on arrival and randomly distributed into the pens with an initial stocking density of 11 birds per pen. The birds had free and continuous access to feed and water throughout the trial. The temperature at pen level was monitored daily and maintained to meet Aviagen recommendations for spot brooding, starting at 34°C at chick arrival and gradually lowered to 20°C by day 25 of age. The lighting schedule was 23L:1D for the first 7 d and was amended to 18L:6D for the course of the trial, while light intensity at pen level ranged from 80 to 100 lux.

Birds were weighed at arrival (day 0), before treatment allocation (day 8), and then twice per week until the end of the trial. After weighing the birds on day 8, the stocking density was reduced from 11 to 10 birds per pen. Chicks were then distributed between pens so that there were no significant differences in the mean BW of each treatment. Pen feed intake was measured from day 0 to day 8, and then daily until the conclusion of the experiment on day 36.

### Sampling

On day 15, 22 and 36 of age, 2 birds per pen with a BW close to the pen average were culled by intravenous lethal injection with sodium pentobarbital (Euthatal, Merial Harlow, United Kingdom). After euthanasia, the full GIT was removed and the small intestine was isolated from the point of entry of the bile ducts to the ileocecal junction. The jejunum was isolated, weighed, and the length was quickly recorded before digesta was collected from the upper one-third of the jejunum for viscosity analysis. The empty carcass weight was obtained by weighing the carcass with the GIT removed. The GIT was then separated into individual components: crop, proventriculus, gizzard, duodenum, jejunum, ileum, ceca, and colon. The length of the duodenum and ileum were recorded, and the components of the GIT were weighed full. After this, the crop, gizzard, duodenum, jejunum, and ileum were carefully emptied of their contents with gentle finger stripping to obtain empty weights.

### Feed Analysis

#### Fibre Analysis

We measured all classical descriptors of feed bulk used traditionally in livestock research. Some of these descriptors are highly correlated as they essentially measure the same bulk characteristics through different measurements (Brachet et al., 2015). Samples of all feeds were analyzed for crude fibre (test method: Commission Regulation (EC) No.152/2009), acid detergent fibre (test method: AOAC 973.18-1977), neutral detergent fibre (test method: AOAC 2002.04-2005), acid detergent lignin (test method: AOAC 973.18-1977), insoluble dietary fibre, soluble dietary fibre, and total dietary fibre (test method: AOAC 2011.25-2011) at a UKAS-accredited commercial laboratory to the internationally recognized standard for competence (Sciantec Analytical Services, Cawood, UK).

#### Water-Holding Capacity

Analysis was performed in triplicate, using an adaptation of the Robertson and Eastwood (1981) method. A 1 g feed sample was soaked in 250 mL of H_2_O at room temperature for 24 h. Subsequently, the samples were filtered through a Whatman no. 1 filter paper and the wet weight of the samples recorded, before the samples were placed into an oven at 105°C overnight and the dry weight was recorded. Water-holding capacity was then calculated as g of water/g of DM.

#### Feed Density

Feed density was determined in triplicate by the method described by Kyriazakis and Emmans (1995). First, 100 mL of distilled water at 37°C was placed in a 250 mL flask and a 50 g sample of feed was added. After mixing, a further 50 mL of water was added, and the contents allowed to equilibrate for 15 min before a final 50 mL of water was added. The sample was left to equilibrate for a further 15 min before the flask was filled to volume with water with a burette. The total amount of water contained in the flask was subtracted from 250 mL.

### Sample Analysis

#### Viscosity

Jejunal digesta samples were thawed in a water bath at 40°C for 15 min. After that, the samples were vortexed for 5 s and then centrifuged at 10,000 RPM at 20°C degrees for 10 min. After centrifugation, the supernatant was carefully removed and pipetted into a 2 mL Eppendorf tube. The tubes were then placed in a water bath at 40°C for 5 min. Finally, 0.5 mL of the supernatant was pipetted into the cone of a Brookfield Model DV-III digital Rheometer (Brookfield Engineering Laboratories, Corp., Stoughton, MA) with a CPE-40 spindle viscosity measurement fitted with a circulating water bath. Readings were obtained at 6 RPM, with a shear rate of 42.5/s at 40°C. Viscosity measurements are reported in centipoise (Cps).

### Calculations and Statistics

Pen average daily energy intake (ADEI, kcal/d) was calculated by multiplying pen ADFI (g/d) by the calculated AME content of the feed. ADFI was calculated over weekly periods, as well as over the whole experimental period (day 8–36). Average daily energy intake, average daily gain (ADG), and feed conversion ratio (FCR) were also calculated over the whole period. To account for *a priori* differences in growth rate due to feed composition performance data were scaled relative to either the mean weekly BW or the mean BW over the whole experimental period (day 22) (Whittemore et al., 2001b). Furthermore, when ADFI was calculated over weekly periods, the data were scaled relative to the mean BW of each week. Organ measurements were expressed relative to the empty carcass weight (ECW) of the bird (g/kg BW) to account for the differences in performance (Oikeh et al., 2019).

All statistical analysis was performed in the nlme package in R (Team, 2013) using its lm and anova functions (Pinheiro et al., 2014). All data were analyzed with the GLM procedure with feed as a fixed factor. Additional polynomial contrasts were performed on both performance data and organ measurements to study the linear and quadratic responses to diet dilution for OH and SBP separately. For all statistical procedures, the normality of the residuals was assessed with qq-plots and the Shapiro-Wilk test. When significant differences were detected, treatment means were separated and compared by the Tukey's multiple comparison test. Significance was determined at *P* < 0.05. Data are presented as model-predicted least square means with their pooled SEM.

## Results

### Feed Analysis

All measured characteristics of feed bulk increased with diet dilution in comparison with the B feed, with the exception of the WHC of OH15. The crude fibre, neutral detergent fibre, acid detergent fibre, and insoluble dietary fibre were greater in the OH series than in the corresponding dilution of the SBP series. The acid detergent fibre content and insoluble dietary fibre were greater in SBP15 than in OH15, but the fibre values were greater in the OH30 and OH45 than the SBP30 and SBP45 feeds, respectively. The WHC, feed density, and soluble dietary fibre were greater in SBP feeds than in OH feeds.

### Feed and Calculated Energy Intake

**Figure 1:**
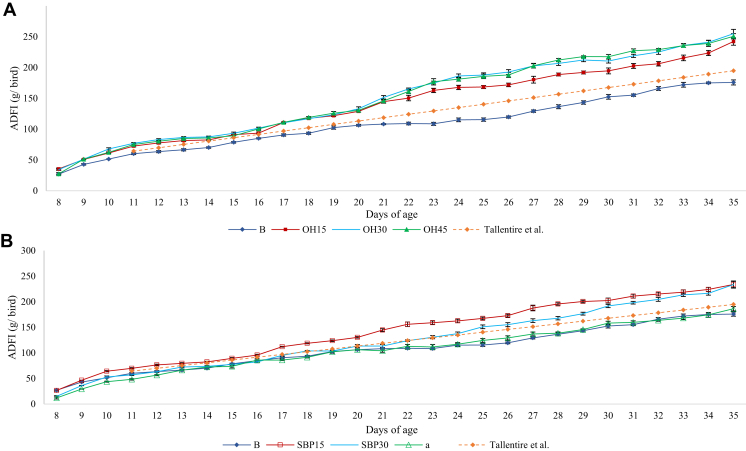
Average daily food intake (ADFI; g/bird) of broiler chickens given access to a basal food (B), or the basal food diluted with 15, 30, or 45% of either a) oat hulls (OH) or b) sugar beet pulp (SBP) from day 8 to 35 of age, and the potential average daily feed intake defined by the apparent biological limit suggested by Tallentire et al. (2018).

The progression of daily feed intake from day 8 to 35 of age is shown in Figures 1A, 1B. The birds offered the OH series or SBP15 feeds were able to increase feed intake above that of the B birds throughout the experiment (*P* < 0.05). By contrast, SBP30 birds were only able to increase their feed intake above that of the B birds from day 22 onward (*P* < 0.05), and the SBP45 birds maintained a similar feed intake to the B birds throughout the experiment (*P* > 0.05).

ADFI, ADEI, and scaled ADFI calculated over the whole period are presented in Table 2. There were no significant differences in ADFI between the birds offered the B and SBP45 feeds (*P* > 0.05). The ADFI of birds on the OH dilution series was significantly greater than that of the birds on the SBP30 and SBP45. The ADFI of the OH series increased linearly as diet dilution increased (*P* < 0.01). Furthermore, quadratic effects of OH and SBP dilutions were observed in ADFI (*P* < 0.01). ADEI of birds offered OH45, SBP30, and SBP45 was significantly lower than B (*P* < 0.01). Quadratic and linear effects of OH and SBP dilution were observed in ADEI (*P* < 0.01). There were no significant differences in ADEI between birds offered OH15, OH30, or B (*P* > 0.05), whereas the SBP15 birds significantly increased ADEI compared with B (*P* < 0.01). When ADFI was scaled to BW, there was a linear increase in scaled ADFI with diet dilution in both the OH and SBP series; quadratic effects were also observed for the SBP dilution (*P* < 0.01). Scaled ADFI was greater in SBP birds than the OH15, OH30, and the B feeds (*P* < 0.05).

**Table 2:** The average daily food (ADFI, g/d), average daily gain (ADG, g/d), and feed conversion ratio (FCR) of broiler chickens given access to a basal food, or the basal food (B) diluted with 15, 30, or 45% of either oat hulls (OH) or sugar beet pulp (SBP) from day 8 to 36 of age.

Treatment	ADFI (g/d)	ADEI (kcal/d)	ADFI/BW (g/kg BW/d)	ADG/BW (g/kg BW/d)	FCR
B	106^a^	329^c^	109^a^	78.3^b,c^	1.32^a^
OH15	135^b,c^	344^c^	122^b^	94.5^f^	1.39^a,b^
OH30	140^c^	323^c^	130^b^	92.7^e,f^	1.53^b,c^
OH45	142^c^	283^b^	144^c^	83.6^c,d^	1.71^c,d,e^
SBP15	138^c^	372^d^	129^b^	86.3^d,e^	1.59^c,d^
SBP30	126^b^	290^b^	148^c^	73.1^b^	1.73^d,e^
SBP45	106^a^	204^a^	148^c^	53.1^a^	1.87^e^
SEM	2.4	7.1	2.5	1.87	0.045
Probabilities					
Feed	<0.001	<0.001	<0.001	<0.001	<0.001
Linear OH	<0.001	<0.001	<0.001	0.341	<0.001
Linear SBP	0.422	<0.001	<0.001	<0.001	<0.001
Quadratic OH	<0.001	0.001	0.994	<0.001	0.572
Quadratic SBP	<0.001	<0.001	<0.001	<0.001	0.483

^a–f^Means within a column that do not share a common superscript are significantly different (*P* < 0.05).

ADFI and ADG scaled relative to body weight (BW) at day 22 (mean point of experimental period; g/kg BW/d) are also given. Data are presented as LS means and SEM.

Abbreviations: ADFI, average daily food intake; ADG, average daily gain; FCR, food conversion ratio.

**Figure 2:**
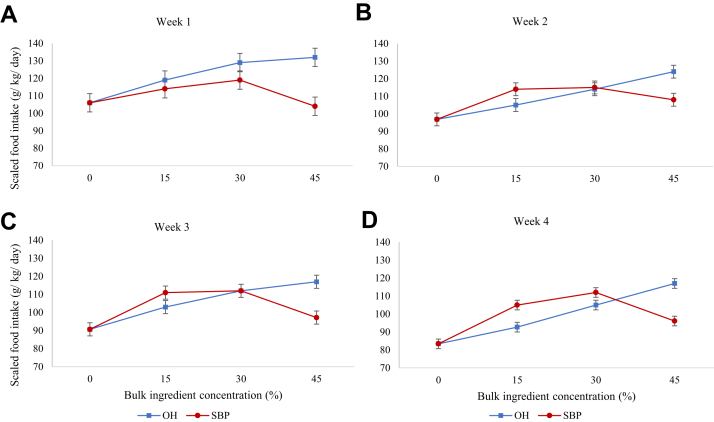
Average daily food intake of broiler chickens for each experimental week, expressed relative to the average body weight of each week (g/kg/d), when offered foods which were diluted with 0, 15, 30, or 45% of either oat hulls (OH) or sugar beet pulp (SBP); (A) week 1; (B) week 2; (C) week 3; (D) week 4. Data are presented as LS means and SEM.

During each of the 4 weekly periods, the birds offered the OH series showed a linear increase in scaled ADFI as dilution increased (Figure 2). Furthermore, there was a quadratic effect of OH dilution in weeks 1 and 3 (*P* < 0.05). The OH birds consumed significantly more feed per unit of BW than B birds throughout the experiment (*P* < 0.05). This was not the case for birds on the SBP series. During week 1, there was no significant difference in the scaled ADFI of birds offered B, SBP15, and SBP45 feeds (*P* > 0.05). Meanwhile the birds offered SBP30 consumed significantly more than those offered B (*P* < 0.05); as a consequence, there was a quadratic effect on scaled ADFI during week 1 (*P* < 0.05). However, during weeks 2 to 4 there was a linear and quadratic effect of SBP dilution on scaled ADFI (*P* < 0.05). During these periods, SBP45 birds increased their scaled ADFI above that of the B birds, so that all of the diluted treatments consumed significantly more than the B birds throughout the rest of the experiment (*P* < 0.05).

### Average Daily Gain and FCR

**Figure 3:**
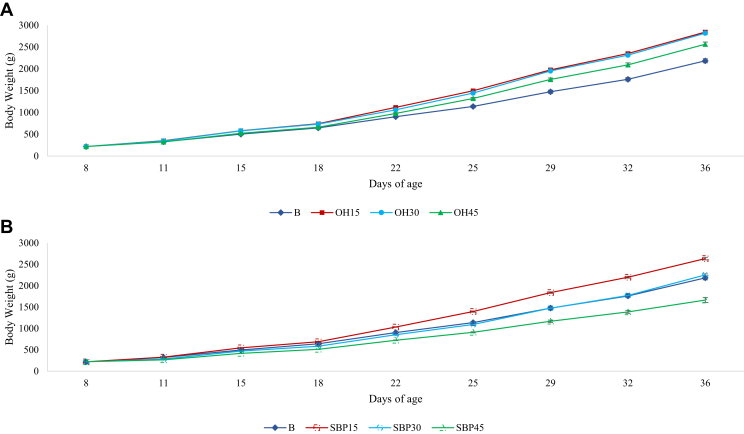
The progression of body weight (g) from day 8 to 36 of broilers chickens given access to a basal food (B), or the basal food diluted with 15, 30, or 45% of either A) oat hulls (OH) or B) sugar beet pulp (SBP).

The progression of body weight from days 8 to 36 of age is shown in Figures 3A, 3B. Over the whole experimental period, ADG relative to BW of the birds offered OH15, OH30, and SBP15 was significantly greater than B (*P* < 0.05) (Table 2). Although there was a linear decrease in relative ADG of the SBP birds (*P* < 0.01), only the performance of birds offered SBP45 was significantly reduced compared with B (*P* < 0.05). Moreover, a quadratic effect was observed for both the OH and SBP series in relative ADG (*P* < 0.01). FCR increased linearly with diet dilution in both the OH and SBP series (*P* < 0.01). There was no significant difference in FCR (Table 2) over the whole experimental period between birds on the B and OH15 feeds (*P* > 0.05). However, all remaining treatments significantly increased FCR compared with B (*P* < 0.05).

### Organ Measurements and Viscosity

Empty carcass weights and organ measurements scaled relative to ECW from day 15, day 22, and day 36 are presented in Table 3, Table 4, Table 5, respectively. There were significant linear and quadratic effects of SBP dilution on ECW at all slaughter points (*P* < 0.01). While there were significant quadratic effects of OH dilution on ECW at all slaughter points, a linear effect on ECW was only observed with OH dilution on day 36 (P < 0.01). On day 15 and 22, the ECW of OH15, OH30, and SBP15 birds were significantly greater than B birds, whereas the SBP30 and SBP45 birds were significantly lighter than B birds (*P* < 0.01). On day 36, the ECW was significantly greater in B birds than the SBP series and OH45 birds (*P* < 0.01). There were no significant differences between OH15, OH30, and B birds (*P* > 0.05).

**Table 3:** Organ measurements expressed relative to empty carcass weight (ECW) of birds slaughtered on day 15 of age.

Treatment	ECW (g)	Crop (g/kg ECW)		Proventriculus full (g/kg ECW)	Gizzard (g/kg ECW)		Small intestine (g/kg ECW)		Caeca full (g/kg ECW)	Large intestine full (g/kg ECW)
		Full	Empty		Full	Empty	Full	Empty		
B	402^c^	19.4	7.00^a,b^	8.38^a^	48.2^a^	32.7^a^	89.7^a^	58.0^a^	15.4	9.78^a^
OH15	477^e^	14.5	5.83^a^	8.29^a^	48.3^a^	33.2^a^	89.8^a^	58.6^a^	17.2	8.59^a^
OH30	464^e^	12.3	5.31^a^	7.34^a^	54.1^a,b^	38.1^a,b^	92.5^a^	62.8^a,b^	15.6	9.85^a^
OH45	400^c^	14.8	7.42^a,b^	8.18^a^	65.8^c^	45.6^c^	104^a,b^	67.6^b^	16.7	12.5^a,b^
SBP15	432^d^	12.7	7.56^a,b^	9.84^b^	64.3^b,c^	38.7^a,b^	119^b^	68.8^b,c^	14.8	14.3^b^
SBP30	350^b^	17.2	8.33^b^	9.89^b^	71.3^c,d^	44.0^b,c^	138^c^	76.1^c^	19.3	15.8^b^
SBP45	306^a^	14.6	8.77^b^	10.8^b^	77.5^d^	49.2^c^	154^c^	84.9^d^	19.4	16.6^b^
SEM	7.1	2.96	0.542	0.319	2.44	1.54	3.82	1.83	1.44	0.951
Probabilities										
Feed	<0.001	0.624	<0.001	<0.001	<0.001	<0.001	<0.001	<0.001	0.107	<0.001
Linear OH	0.952	0.558	0.979	0.587	<0.001	<0.001	0.002	<0.001	0.971	0.069
Linear SBP	<0.001	0.838	0.125	<0.001	<0.001	<0.001	<0.001	<0.001	0.021	<0.001
Quadratic OH	<0.001	0.539	0.002	0.358	0.046	0.059	0.122	0.629	0.994	0.111
Quadratic SBP	<0.001	0.871	0.996	0.800	0.191	0.992	0.405	0.943	0.987	0.179

^a–d^Means within a column that do not share a common superscript are significantly different (*P* < 0.05).

Broiler chickens were offered foods diluted with 0 (B), 15, 30, or 45% of either oat hulls (OH) or sugar beet pulp (SBP) from day 8 of age.

Abbreviations: BW, body weight; ECW, empty carcass weight.

**Table 4:** Organ measurements expressed relative to empty carcass weight (ECW) from day 22 of age.

Treatment	ECW (g)	Crop (g/kg ECW)		Proventriculus full (g/kg ECW)	Gizzard (g/kg ECW)		Small intestine (g/kg ECW)		Caeca full (g/kg ECW)	Large intestine full (g/kg ECW)
		Full	Empty		Full	Empty	Full	Empty		
B	782^c^	17.3a	6.15a	7.37^b,c^	36.4^a^	23.1^a^	80.3^a^	48.5^a,b^	17.1^b,c^	10.8^a,b^
OH15	957^f^	18.7^a^	7.01^a,b^	6.06^a^	37.3^a^	26.1^a^	75.8^a^	46.7^a^	13.9^a,b^	7.50^a^
OH30	879^e^	43.1^b^	8.80^b^	6.20^a,b^	47.2^b^	32.5^b^	84.8^a,b^	53.0^b,c^	13.6^a,b^	8.82^a^
OH45	810^c,d^	30.3^a,b^	7.84^a,b^	6.55^a,b^	49.6^b^	36.4^c^	94.8^b^	56.2^c,d^	13.2^a,b^	9.59^a,b^
SBP15	857^d,e^	17.9^a^	6.84^a,b^	7.87^c^	52.1^b^	30.2^b^	111^c^	59.2^d^	10.9^a^	12.7^b^
SBP30	666^b^	16.3^a^	7.93^a,b^	10.0^d^	66.7^c^	36.8^c^	133^d^	66.1^e^	13.4^a,b^	16.6^c^
SBP45	524^a^	27.5^a,b^	8.43^a,b^	10.2^d^	69.7^c^	38.3^c^	172^e^	75.1^f^	19.5^c^	21.1^d^
SEM	14.9	4.12	0.571	0.282	1.47	0.94	2.49	1.27	0.95	0.893
Probabilities										
Feed	<0.001	<0.001	<0.001	<0.001	<0.001	<0.001	0.036	<0.001	<0.001	<0.001
Linear OH	0.999	0.019	0.079	0.014	<0.001	<0.001	<0.001	<0.001	0.041	0.886
Linear SBP	<0.001	0.260	0.008	<0.001	<0.001	<0.001	<0.001	<0.001	0.140	<0.001
Quadratic OH	<0.001	0.374	0.417	<0.001	0.887	0.922	<0.001	0.074	0.403	0.024
Quadratic SBP	<0.001	0.413	0.997	0.947	0.005	0.049	0.569	0.934	<0.001	0.512

^a–f^Means within a column that do not share a common superscript are significantly different (*P* < 0.05).

Broiler chickens were offered foods diluted with 0% (B), 15, 30, or 45% of either oat hulls (OH) or sugar beet pulp (SBP).

Abbreviations: BW, body weight; ECW, empty carcass weight.

**Table 5:** Organ measurements expressed relative to empty carcass weight (ECW) from day 36 of age.

Treatment	ECW (g)	Crop (g/kg ECW)		Proventriculus full (g/kg ECW)	Gizzard (g/kg ECW)		Small intestine (g/kg ECW)		Caeca full (g/kg ECW)	Large intestine full (g/kg ECW)
		Full	Empty		Full	Empty	Full	Empty		
B	2,518^d^	7.44^a^	4.25^a^	1.68^a^	24.5^a^	17.4^a^	47.1^a,b^	32.2^a,b^	11.2^a^	8.24^a,b^
OH15	2,610^d^	13.3^a^	4.90^a^	1.47^a^	26.4^a^	18.2^a^	44.4^a^	29.2^a^	8.69^a^	7.83^a,b^
OH30	2,498^d^	8.75^a^	5.05^a^	1.47^a^	29.0^a,b^	19.6^a^	47.1^a,b^	31.9^a,b^	10.4^a^	6.64^a^
OH45	2,211^c^	9.71^a^	4.83^a^	1.47^a^	34.0^b^	23.9^b^	55.5^b,c^	35.0^b,c^	9.93^a^	9.00^a,b^
SBP15	2,262^c^	8.63^a^	5.40^a^	1.73^a^	34.0^b^	20.7^a,b^	62.7^c^	38.8^c^	9.33^a^	11.5^b^
SBP30	1,876^b^	12.0^a^	6.24^a^	2.23^b^	49.4^c^	24.0^b^	87.3^d^	46.7^d^	11.6^a^	16.3^c^
SBP45	1,301^a^	29.7^b^	9.69^b^	2.70^c^	71.3^d^	30.4^c^	137^e^	58.4^e^	15.4^b^	21.8^d^
SEM	34.8	2.59	0.715	0.066	1.51	1.17	3.01	1.06	1.079	1.277
Probabilities										
Feed	<0.001	<0.001	<0.001	<0.001	<0.001	<0.001	<0.001	<0.001	<0.001	<0.001
Linear OH	<0.001	0.994	0.894	0.176	<0.001	<0.001	0.001	0.039	0.919	0.987
Linear SBP	<0.001	<0.001	<0.001	<0.001	<0.001	<0.001	<0.001	<0.0001	0.007	<0.001
Quadratic OH	<0.001	0.529	0.871	0.213	0.224	0.041	0.002	0.004	0.438	0.203
Quadratic SBP	<0.001	<0.001	0.409	0.004	0.016	0.432	<0.001	0.104	0.017	0.757

^a–e^Means within a column that do not share a common superscript are significantly different (*P* < 0.05).

Broiler chickens were offered foods diluted with 0% (B), 15, 30, or 45% of either oat hulls (OH) or sugar beet pulp (SBP).

Abbreviations: BW, body weight; ECW, empty carcass weight.

There were no significant differences in the relative full crop and relative full ceca weights on day 15 between any of the dietary treatments (*P* > 0.05). On day 15, there was a significant quadratic effect of OH dilution on the relative weight of the empty crop (*P* < 0.01). On day 22, there was a significant linear effect of OH dilution on the relative full crop weight (*P* < 0.05) and a significant linear effect of SBP dilution on the relative empty crop weight (*P* < 0.01); whereas, on day 36 there were significant linear effects of SBP dilution on the relative full and empty crop weight (*P* < 0.01), and a significant quadratic effect of SBP dilution on the relative full crop weight (*P* < 0.01). On day 15, there were no significant differences in relative full and empty crop weights between the B birds and the birds given the diluted feeds (*P* > 0.05), whereas on day 22, the relative weight of the full and empty crop was significantly increased in OH30 birds compared with B birds (*P* < 0.01). The relative full and empty crop weight of birds on day 36 were significantly increased in SBP45 birds compared with all other treatments (*P* < 0.01).

There was a significant linear increase in relative weight of the full proventriculus at all slaughter points for SBP birds (*P* < 0.01). Relative proventriculus full weight was significantly greater in SBP birds compared with birds offered the OH series or the B feed at all slaughter points (*P* < 0.01). The exception to these effects was the significantly lower full proventriculus weight of OH15 compared with B birds on day 22.

There were significant linear effects of OH and SBP dilution on relative full and empty gizzard weight at all slaughter points (*P* < 0.01); furthermore, significant quadratic effects of SBP dilution were observed in relative full and empty gizzard weight on day 22, and full gizzard weight on day 36 (*P* < 0.01). On day 36, there was also a significant quadratic effect of OH dilution on the relative empty gizzard weight (*P* < 0.01). Relative full gizzard weight was significantly increased in SBP and OH45 birds compared with B birds at all slaughter points (*P* < 0.01). Relative empty gizzard weight was also significantly greater in SBP and OH45 birds at all slaughter points (*P* < 0.01), with the exception of SBP15 on day 15 and day 36 (*P* > 0.05).

There were significant linear effects caused by feed dilution on the relative full and empty weights of the small intestine from birds on both the OH and SBP series at all slaughter points (*P* < 0.05). There were quadratic effects of feed dilution on the relative full weight of the small intestine from birds on the OH series on day 22 and day 36 (*P* < 0.01). Furthermore, on day 36 there were quadratic effects of SBP dilution on relative full small intestine weight and quadratic effects of OH dilution on relative empty weight of the small intestine (*P* < 0.01). As a consequence, there were no significant differences in the relative weight of the full and empty small intestine of birds offered the B feed or the OH series (*P* > 0.05) at any of the slaughter points, with the exception of the OH45 birds, where relative empty small intestine weight was increased at day 15 and day 22, and relative full small intestine weight increased at day 22 (*P* < 0.01). By contrast, the full and empty relative weights of the small intestine were significantly greater in SBP birds than in B at all slaughter points (*P* < 0.01).

There were quadratic effects of OH dilution on the relative lengths of the small intestine (Supplementary Table 1) on day 15 and day 22 (*P* < 0.01) and linear effects of OH dilution on day 36 (*P* < 0.01); whereas linear effects of SBP dilution were observed on day 15 (*P* < 0.01) and linear and quadratic effects of SBP dilution on the relative lengths of the small intestine (*P* < 0.01). There were no significant differences in the relative lengths of the sections of the small intestine between the birds offered the B feed and the OH series at all slaughter points (*P* > 0.05), with the exception of the relative jejunum length of OH45 birds, which was significantly longer than that of the B birds on day 36 (*P* < 0.01). By contrast, the SBP series yielded significantly longer intestinal segments than B at all slaughter points (*P* < 0.01), with the exception of SBP15 birds at day 22 (duodenum, jejunum, and ileum) and day 36 (duodenum), which were not significantly different from B birds (*P* > 0.05).

There were no significant differences in relative full ceca weight between all dietary treatments at day 15 (*P* > 0.05), although there was a linear effect of SBP dilution (*P* < 0.05). Relative full ceca weight on day 22 linearly increased with OH dilution (*P* < 0.05), but were not statistically different from B (*P* > 0.05). Furthermore, there was a quadratic effect of SBP dilution on relative full ceca weight on day 22 (*P* < 0.05), where there was a significant reduction in relative ceca weight of SBP15 birds compared with B birds (*P* < 0.01). There were linear and quadratic effects of SBP dilution on the relative full ceca weight on day 36 (*P* < 0.05), which was significantly greater in SBP45 birds than in all other treatments (*P* < 0.01).

There was a quadratic effect of OH dilution on the relative full large intestine weight at day 22 (*P* < 0.05), although there were no significant differences between B and OH birds at any of the slaughter points (*P* > 0.05). The relative full large intestine weight linearly increased with SBP dilution at all slaughter points (*P* < 0.01). This led to significantly greater relative full large intestine weights at all slaughter points for SBP birds than for B birds (*P* < 0.01), with the exception of the SBP15 birds at day 22 and 36 (*P* > 0.05).

The effect of diet dilution on jejunal viscosity (cP) was similar across slaughter points, so we report day 36 effects only. Jejunal viscosity increased linearly with SBP dilution: SBP15, 2.82 (CI 2.61–3.03); SBP30, 3.57 (CI 3.31–3.84); SBP45, 4.11 (CI 3.86–4.35) (*P* < 0.01). This was not the case for OH dilution, as there were no significant differences between the OH and B birds: OH15, 1.98 (CI 1.69–2.27); OH30, 2.05 (CI 1.83–2.27); OH45, 2.37 (CI 2.14–2.59); B, 2.18 (CI 1.95–2.40).

## Discussion

The experiment was designed to test the ability of a modern fast-growing broiler to cope with increasing levels of diet dilution with bulky ingredients. We formulated the feeds so that energy was the first limiting nutrient resource because the prevailing view is that animals eat for the first limiting nutrient resource in their feed (Emmans, 1986; Gous, 2007). The CP content of the basal feed was slightly above Ross 308 nutrient recommendations (Aviagen Ltd., Edinburgh, UK). By doing this, we aimed to avoid deficiencies in specific amino acids, such as threonine, for which demand is increased to cope with an increase in epithelial cell turnover as a result of the high dietary fibre contents of the diluted feeds (Montagne et al., 2003; Bortoluzzi et al., 2018). Based on this assumption, we were able to investigate broiler capacity for bulk as the energy content of the feed was reduced, whereas the bulk content of the feed increased. To test our hypothesis, OH and SBP were selected as diluents because of distinct differences in their bulk properties (such as fibre content, solubility, WHC, and feed density) which were expected to affect feed intake and GIT development in different ways (Jiménez-Moreno et al., 2013b; Jørgensen et al., 1996b; Savory et al., 1996).

The suggestion has been put forward that modern strains of broilers are no longer able to regulate their feed intake when they are given access to feeds of different energy contents (see reviews by Classen (2017) and Mbajiorgu et al. (2011)). Our results are inconsistent with this suggestion. For the OH series, birds were able to increase linearly their feed intake, in a manner directly related to the linear reduction in energy intake. For example, the feed energy content of OH30 (2,273 kcal/kg feed) was 26% lower than the B feed (3,081 kcal/kg feed), which led OH30 birds to increase ADFI by 22% compared with B birds. This resulted in a similar ADEI by birds in these 2 treatments. The energy content of the OH30 feed was lower than in studies which did not observe an increase in feed intake (Plumstead et al., 2007; Latshaw, 2008; Delezie et al., 2010; Li et al., 2010; Krás et al., 2013) and experiments where feed intake was increased (Deaton and Lott, 1985; Dozier et al., 2006; Leeson et al., 1996a; Linares and Huang, 2010). We suggest, therefore, that provided that the bulkiness of the feeds does not limit the feed intake of the birds, birds will respond to the dilution of the energy content of the feed in a manner consistent with the principle of the regulation of feed intake (Emmans, 1986; Gous, 2007, 2013). Whether birds would continue to adapt to the feed energy content, will depend on the ability of their digestive tract to adapt to the bulkiness that accompanies such a dilution.

Tallentire et al. (2018a) suggested that modern broilers are reaching the limits of feed intake. The highest feed intake previously seen by broilers has been observed by Leeson et al. (1996a), who showed that broilers were able to increase feed intake by 25% without negatively affecting performance when offered feeds diluted with 3.75% each of OH and sand, compared to birds offered a basal feed. Tallentire et al. (2018a) assumed that this is the maximum capacity of the GIT for intake in broilers. On the basis of this, they then estimated how much the feed intake capacity of modern strains could increase. We compared this estimate with our data in Figure 1, which shows the ADFI (g/d) from our experiment plotted alongside the estimated daily feed intake calculated by Tallentire et al. (2018a). Over the period of 22 to 36 d, when it may be assumed that the adaptation of the GIT had reached equilibrium (see the following), the ADFI of the birds on OH30 was 101 g. This is 20% higher than the “maximum” feed intake assumed by Tallentire et al. (2018a) over the same live weight range for this broiler strain.

We hypothesized that broilers offered feeds diluted with SBP will be limited in their performance further than those offered feeds diluted with OH due to differences in the bulk properties of the 2 ingredients. When the performance of the birds over the whole experimental period was considered, all birds on the diluted diets had a higher ADFI than the birds on the B feed, with the exception of the birds on SBP45. Given that dilution of the B feed with the bulky ingredients resulted in reduction of the energy content of the diets, birds on OH45, SBP30, and SBP45 consumed less ADEI than birds on B throughout the experiment and as a consequence their growth performance was penalized. One therefore, might be tempted to suggest that these 3 feeds were limiting energy intake through their bulkiness. However, the long-term picture (35 d) is likely to be masking 2 separate effects: 1) the relationship between ADFI and BW (Whittemore et al., 2003a) and 2) any adaptation that might be occurring over time on the bulky feeds (Zubair and Leeson, 1996; Tsaras et al., 1998; Whittemore et al., 2001a). For this reason, we scaled ADFI per unit of BW and then analyzed the scaled ADFI for each of the experimental weeks (week 1–4). These calculations suggest that while scaled ADFI might have been limited in some diets during weeks 1 and 2, by week 4 it was only the SBP45 feed that appeared to be limiting scaled ADFI (and thusly the energy intake) of the birds: the scaled ADEI of week 4 was 187 (SE 2.85) and 251 (SE 3.37) (kcal/kg BW/d) on diets SBP45 and B, respectively (*P* < 0.01). As a consequence of the increased scaled ADFI, the birds offered the diluted feeds (other than SBP45) were able to maintain performance to that of the birds given the B feed over the whole experimental period. We therefore partially fulfilled the objective of reducing performance in the SBP birds further than the OH birds, although only the SBP45 birds were limited.

As expected and consistent with the ADFI, the relative weights of the components of the GIT increased as the amount of the bulky ingredients in the feeds increased, thus demonstrating the great phenotypic plasticity of the broiler GIT (Klasing, 1999). For the OH series, the increase in the relative weights GIT components was confined to the gizzard and small intestine. Gizzard development was more pronounced in the SBP series than the OH series, which is inconsistent with studies that have shown the converse and their authors ascribe the effect to the greater insoluble fibre content of OH (Jiménez-Moreno et al., 2013b). Svihus (2011) suggested that the grinding capability of the gizzard may be limited when birds are fed high fibre feeds. On the contrary, our results show that the gizzard responded linearly to diet dilution at each of the slaughter points and therefore was not limited in its development or responsible for limiting feed intake. This is consistent with other authors who also observed a linear increase in gizzard development when offering feeds with increasing levels of dietary fibre (Summers and Leeson, 1986; Jiménez-Moreno et al., 2011; Miya et al., 2019; Okrathok and Khempaka, 2020).

Typically, gizzard development and retention time have been investigated in relation to insoluble fibre sources (Amerah et al., 2009; Sacranie et al., 2012), which are often offered in mash form (Adibmoradi et al., 2016; Abdollahi et al., 2019) or crumble (Svihus et al., 2002; Svihus et al., 2004; Hetland et al., 2005). Furthermore, Jiménez-Moreno et al. (2019) showed that the effect of diet dilution with insoluble fibre sources on GIT development is greater when the feeds are offered as mash rather than pellets, with the exception of the crop. It is therefore possible that the differences in gizzard development between our experiment and that of Jiménez-Moreno et al. (2013b) are due to differences in feed form. To that end, it is important that feed form should be considered when assessing the adaptation and capacity of the GIT to bulky feeds.

The remaining organ measurements from our experiment were greater in the SBP rather than OH series, consistent with the findings of Jiménez-Moreno et al. (2013b). For the OH series, the increase in the relative GIT weights was confined to the gizzard and small intestine. For the SBP series, the increase in the relative weights was also extended to the proventriculus and the large intestine. Sugar beet pulp is rich in soluble nonstarch polysaccharides such as pectin and therefore has a high WHC, which results in wetter faeces (Mossami, 2011) and this physical distension of the GIT (or bulk increase) is carried throughout the digestive tract (González-Alvarado et al., 2010). Consistent with our results, the relative weight of the proventriculus has been shown to increase when broilers are offered feeds diluted with bulky ingredients (Amerah et al., 2009). However, the role of the large intestine in the accommodation of bulky feeds is often overlooked (Amerah et al., 2009; Jiménez-Moreno et al., 2009; 2013b; Adibmoradi et al., 2016; Abdollahi et al., 2019), although there is some evidence showing an increase in the relative weight of the large intestine when broilers given feeds diluted with pea fibre, wheat bran, or oat bran (Jørgensen et al., 1996a).

The initial reduction in feed intake when an animal is offered a bulky feed, or inability to increase feed intake to maintain energy intake, reflects the fact that the GIT may not have yet fully adapted to the bulky feed (Starck, 1999; Whittemore et al., 2003b). There was some evidence that this was the case for the OH30, OH45, SBP15, and SBP30 feeds. For the first 3 feeds, the change in the rate of ADFI seems to have taken 8 to 10 d, whereas it was around 14 to 16 d for the SBP30. This is also reflected in the weekly scaled ADFI (Figure 2): birds on SBP15 increased their relative ADFI from week 2. It was only by week 4 that the relationship between ADFI on B, SBP15 and SBP30 became linear. Finally, the higher relative ADFI on the SBP as opposed to OH series (with the exception of SBP45) by week 4 is consistent with the lower energy content of the SBP feeds, and the need to further compensate for this. As our experiment was conducted on birds and of a relatively young age (8 d), it is possible that different adaptation responses would have occurred if the birds were offered the bulky feeds at a later age (Mateos et al., 2012). Evidence shows that when broilers are not introduced to the diluted feeds until day 35 of age they are unable to increase feed intake to compensate for the reduction in energy content (Leeson et al., 1996b; Sahraei and Shariatmadari, 2007). This is logical when one considers the developmental plasticity of a newly hatched chick and the dramatic relative increases in GIT development in the first 10 d of life (Sklan, 2001). These suggestions are of relevance when considering the age at which to introduce lower energy or bulky feeds to growing broilers.

The second objective of the experiment was to identify a bulk property that may be used to predict the feed intake of broilers on bulky feeds. As discussed previously, the only feed that appeared to be limiting ADFI throughout the experiment was SBP45; in addition, birds fed the SBP series had greater relative weights of the GIT than birds on the OH series, suggesting that the birds given SBP needed to accommodate a higher bulk intake. Of the several measurements of bulk we analyzed, SBP feeds had a higher WHC, feed density and soluble dietary fibre content than OH feeds at the same level of inclusion. Some of these bulk characteristics are expected to be highly correlated (such as WHC and soluble fibre content), as they relate to the same physicochemical properties (Brachet et al., 2015). Although we did not measure the digestibility of our feeds, other work (Jiménez-Moreno et al., 2013a) has suggested that the digestibility of OH and SBP feeds is similar for the same levels of inclusion. The Kyriazakis group (Kyriazakis and Emmans, 1995; Tsaras et al., 1998; Whittemore et al., 2003b) has suggested that for pigs the WHC of the feeds is a good predictor of scaled feed intake on bulky feeds. The relationship appears to be of the form: scaled ADFI (g/kg/d) = *a* ∗ 1/WHC (g feed/g water). If it is assumed that the same relationship applies for broilers then the value of *a* for the SBP30 and SBP45 feeds would be 700 (SE 10.5) and 889 (SE 11.3), during the last week of the experiment. The critical WHC value of the feed that determines maximum capacity for bulk would be between 4.47 and 6.01 (g/g DM). Clearly this hypothesis needs to be tested on a wider range of feed ingredients and on feeds that would certainly limit the feed intake of the birds throughout the experimental period.

In conclusion, we do not offer support to the suggestion that modern broiler strains are no longer able to control their feed intake when they are given access to feeds of different energy contents (see reviews by Classen (2017) and Mbajiorgu et al. (2011)). Birds responded to the dilution of the basal, high quality feed in a manner expected from the principle of regulation of energy intake. In fact, the extent of the increase in feed intake is well beyond the increases previously seen in any experiment addressing the capacity of broilers for bulky feeds (Dozier et al., 2006; Leeson et al., 1996a; Linares and Huang, 2010). Given the fact that our levels of inclusion of OH and SBP in the feeds are well above what one might envisage in practice when incorporating bulky ingredients, the concerns about the energy content of the modern broiler feeds Linares and Huang, 2010; Gous, 2013; Classen, 2017; Tallentire et al., 2018a) may be unwarranted. We appreciate that we used only one broiler strain in our study (Ross 308), but given its global popularity, some generalization about our findings may be justified. Identifying a property of feed “bulkiness” that will allow the prediction of feed intake of broilers given bulky feeds remains a challenge.
