# Supplementary material for: What are the limits to feed intake of broilers on bulky feeds?
Source: Poult Sci. 2020 Nov 19;100(3):100825. doi: 10.1016/j.psj.2020.11.008 (PMC7936167; doi:10.1016/j.psj.2020.11.008)
Supplement: Supplementary_Table [file mmc1.docx]

Table S1. Duodenum, jejunum and ileum lengths expressed relative to empty carcass weight (ECW) of birds slaughtered on day (d) 15, 22 and 36 of age. Broiler chickens were offered foods diluted with 0 (B), 15, 30, or 45% of either Oat Hulls (OH) or Sugar Beet Pulp (SBP) from day 8 of age.

|  | d15 | | | d22 | | | d36 | | |
| --- | --- | --- | --- | --- | --- | --- | --- | --- | --- |
|  | Duodenum length (cm/ kg ECW) | Jejunum length (cm/ kg ECW) | Ileum length (cm/ kg ECW) | Duodenum length (cm/ kg ECW) | Jejunum length (cm/ kg ECW) | Ileum length (cm/ kg ECW) | Duodenum length (cm/ kg ECW) | Jejunum length (cm/ kg ECW) | Ileum length (cm/ kg ECW) |
| B | 48.8^ab^ | 133^a^ | 126^ab^ | 27.4^ab^ | 83.8^b^ | 76.4^ab^ | 11.7^ab^ | 29.7^a^ | 29.4^ab^ |
|  |  |  |  |  |  |  |  |  |  |
| OH15 | 44.5^a^ | 125^a^ | 112^a^ | 24.9^a^ | 70.6^a^ | 66.0^a^ | 11.3^a^ | 31.6^a^ | 28.5^a^ |
| OH30 | 42.7^a^ | 126^a^ | 115^ab^ | 27.8^ab^ | 76.0^ab^ | 70.0^ab^ | 12.0^ab^ | 33.2^a^ | 31.0^ab^ |
| OH45 | 49.4^ab^ | 140^a^ | 128^b^ | 30.4^b^ | 81.1^b^ | 75.8^ab^ | 13.4^bc^ | 39.3^b^ | 34.1^bc^ |
|  |  |  |  |  |  |  |  |  |  |
| SBP15 | 49.7^ab^ | 136^a^ | 124^ab^ | 29.2^ab^ | 79.4^ab^ | 79.0^b^ | 12.7^ab^ | 38.4^b^ | 35.8^c^ |
| SBP30 | 56.6^bc^ | 156^b^ | 144^c^ | 36.1^c^ | 95.7^c^ | 95.6^c^ | 15.0^c^ | 45.7^c^ | 42.7^d^ |
| SBP45 | 59.8^c^ | 170^b^ | 157^c^ | 44.9^d^ | 129^d^ | 121^d^ | 22.6^d^ | 64.1^d^ | 63.2^e^ |
|  |  |  |  |  |  |  |  |  |  |
| SEM | 2.11 | 3.6 | 3.5 | 1.05 | 2.56 | 2.38 | 0.48 | 1.03 | 1.29 |
|  | *Probabilities* | | | | | | | | |
| Diet | <0.001 | <0.001 | <0.001 | <0.001 | <0.001 | <0.001 | <0.001 | <0.001 | <0.001 |
| Linear OH | 1.000 | 0.424 | 0.888 | <0.001 | <0.001 | <0.001 | <0.001 | <0.001 | <0.001 |
| Linear SBP | <0.001 | <0.001 | <0.001 | 0.006 | 0.988 | 0.992 | 0.025 | <0.001 | 0.002 |

^a-f^ Means within a column that do not share a common superscript are significantly different (*P* < 0.05)
Abbreviations: ECW, empty carcass weight.
